# How mutations affect function

**DOI:** 10.7554/eLife.99991

**Published:** 2024-06-28

**Authors:** Thomas Kuhlman

**Affiliations:** 1 Department of Physics and Astronomy, University of California, Riverside Riverside United States

**Keywords:** SARS-CoV-2, genotype-phenotype relationship, biophysical fitness landscape, mutant spectrum, intrinsically disordered protein, protein evolution, Viruses

## Abstract

A new study reveals how naturally occurring mutations affect the biophysical properties of nucleocapsid proteins in SARS-CoV-2.

**Related research article** Nguyen A, Zhao H, Myagmarsuren D, Srinivasan S, Wu D, Chen J, Piszczek G, Schuck P. 2024. Modulation of biophysical properties of nucleocapsid protein in the mutant spectrum of SARS-CoV-2. *eLife* **13**:RP94836. doi: 10.7554/eLife.94836.

The COVID-19 pandemic has inflicted enormous societal, economic, and personal costs to populations around the world. It has resulted in hundreds of millions of infections, millions of deaths as well as lingering effects such as persistent symptoms and long COVID. Combatting the virus that causes COVID-19, known as SARS-CoV-2, as well as other viruses that could cause future pandemics, requires an understanding of the basic mechanisms through which viruses evolve and exert their effects.

Every person infected with COVID-19 carries their own pool of viral variants, which each contain a unique set of mutations in their genome. State-of-the-art sequencing technology has allowed an organization known as the Global Initiative on Sharing All Influenza Data to create a SARS-CoV-2 repository, which currently contains around 17 million different viral genome sequences (Elbe and Buckland-Merrett, 2017). This provides unparalleled insight into how mutations correlate with the evolution and emergence of SARS-CoV-2 variants of concern, such as delta and omicron.

However, despite this wealth of available sequencing information, our understanding of how these genetic changes actually impact how variants behave, such as their ability to infect cells and the severity of symptoms they cause, is lacking. Now, in eLife, Peter Schuck and colleagues at the National Institutes of Health – including Ai Nguyen as first author – report how mutations affect a structural protein in SARS-CoV-2 that is responsible for packaging and protecting the viral genome (Nguyen et al., 2024).

Nguyen et al. focused their study on nucleocapsid protein, the most abundant SARS-CoV-2 protein in infected cells, which is typically the molecule probed by at-home tests for the virus (Finkel et al., 2021; Frank et al., 2022). The primary role of the nucleocapsid protein is to package the genomic material of SARS-CoV-2 into the shell of the viral particle. But nucleocapsid protein also binds to many proteins in the host cell, which can modify stress and immune responses that may kill the virus or affect the severity of the infection (Chen et al., 2020; Gordon et al., 2020; Li et al., 2020). These interactions are determined by the structure and biophysical properties of nucleocapsid protein, such as its electrical charge and hydrophobicity.

By studying available genetic sequences of SARS-CoV-2 nucleocapsid proteins, Nguyen et al. found that most mutations lie in intrinsically disordered regions of the protein (Figure 1). These parts of the protein are highly flexible and do not fold into any rigidly defined structure. As such, they are more tolerant to a wider diversity of changes and mutations than other, more highly ordered segments of the protein.

**Figure 1.**
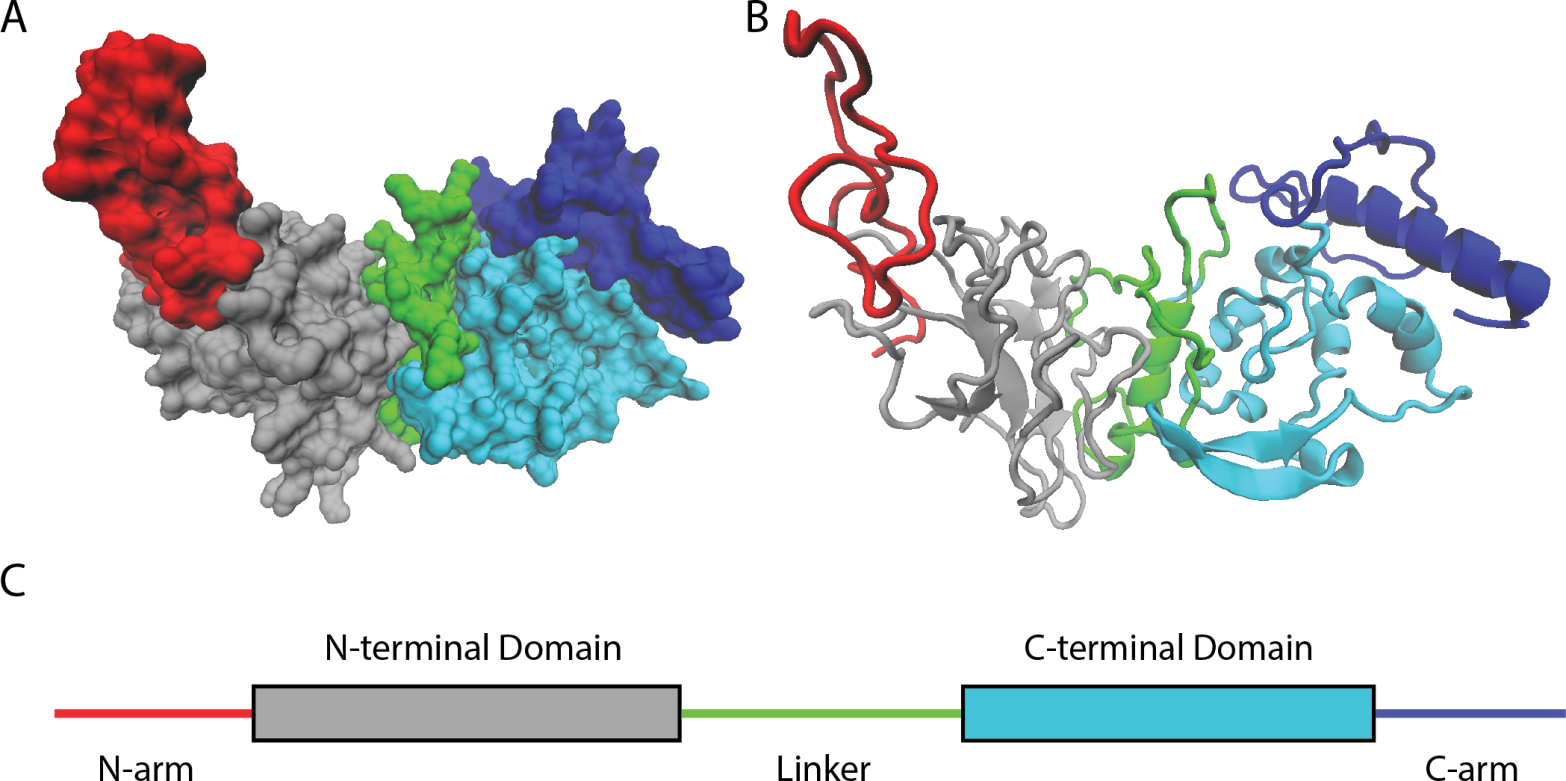
Naturally occurring mutations in the nucleocapsid protein of SARS-CoV-2. (**A**) Space filling, (**B**) ribbon, and (**C**) domain representation of a single SARS-CoV-2 nucleocapsid protein. Nguyen et al. identified that most mutations occur in intrinsically disordered regions of the protein – the N-arm (red), linker (green), and C-arm (blue) – as opposed to more structured regions, such as the N-terminal (grey) and C-terminal (cyan) domains.

Image credit: Atomic coordinates used from Casasanta et al., 2023 and graphics in (**A**) and (**B**) made using Visual Molecular Dynamics (Humphrey et al., 1996).

Nguyen et al. then used computational methods to calculate how the behaviour of nucleocapsid proteins would be expected to change in response to each of the mutations they had detected. This showed that while single mutations in the intrinsically disordered regions cause a broader distribution of effects than those in structured regions of the protein, the biophysical properties of the protein were, surprisingly, largely conserved. Moreover, Nguyen et al. demonstrated that the intrinsically disordered regions show little sequence similarity to related viruses, such as SARS-CoV-1, Middle Eastern Respiratory Syndrome and other coronaviruses. However, the biophysical properties of these regions, such as their polarity and hydrophobicity, were similar across the different viruses.

To investigate further, Nguyen et al. carried out experiments on nucleocapsid proteins which had been purified from SARS-CoV-2 and contained mutations found in several variants of interest. This revealed that when multiple mutations are present, this leads to significant – and sometimes compensatory – biophysical changes. This included changes in the thermal stability of the protein, its ability to self-associate into multi-protein complexes, and how it assembles into larger structures. These effects demonstrate how mutations in intrinsically disordered regions can impact the entire structure and function of a protein. Additionally, this emphasizes why it is difficult to assign variant properties and fitness effects to a single mutation.

The findings of Nguyen et al. suggest that the biophysical properties of intrinsically disordered regions – such as their charge, polarity and hydrophobicity – may contribute to determining the behavior and interactions of nucleocapsid proteins. This could also be the case for other SARS-CoV-2 proteins, as well as proteins in other infectious viruses. These properties may place constraints on the mutations that nucleocapsid proteins can tolerate while still remaining functional. Furthermore, the findings of Nguyen et al. could provide a foundation for understanding how genomic sequencing data can be used to predict the effects of mutations in viruses.
